# SARS-CoV-2 detection using isothermal amplification and a rapid, inexpensive protocol for sample inactivation and purification

**DOI:** 10.1073/pnas.2011221117

**Published:** 2020-09-08

**Authors:** Brian A. Rabe, Constance Cepko

**Affiliations:** ^a^Department of Genetics, Blavatnik Institute, Harvard Medical School, Boston, MA 02115;; ^b^Howard Hughes Medical Institute, Blavatnik Institute, Harvard Medical School, Boston, MA 02115; ^c^Department of Ophthalmology, Blavatnik Institute, Harvard Medical School, Boston, MA 02115

**Keywords:** SARS-CoV-2, diagnostic, RT-LAMP

## Abstract

The current severe acute respiratory syndrome coronavirus 2 (SARS-CoV-2) pandemic has had an enormous impact on society worldwide, threatening the lives and livelihoods of many. The effects will continue to grow and worsen if economies begin to open without the proper precautions, including expanded diagnostic capabilities. To address this need for increased testing, we have developed a sensitive reverse-transcription loop-mediated isothermal amplification (RT-LAMP) assay compatible with current reagents, which utilizes a colorimetric readout in as little as 30 min. A rapid inactivation protocol capable of inactivating virions, as well as endogenous nucleases, was optimized to increase sensitivity and sample stability. This protocol, combined with the RT-LAMP assay, has a sensitivity of at least 50 viral RNA copies per microliter in a sample. To further increase the sensitivity, a purification protocol compatible with this inactivation method was developed. The inactivation and purification protocol, combined with the RT-LAMP assay, brings the sensitivity to at least 1 viral RNA copy per microliter in a sample. This simple inactivation and purification pipeline is inexpensive and compatible with other downstream RNA detection platforms and uses readily available reagents. It should increase the availability of SARS-CoV-2 testing as well as expand the settings in which this testing can be performed.

## Significance

This work describes the optimization of a sample preparation and detection pipeline for a SARS-CoV-2 diagnostic test that is rapid and does not require specialized equipment. This pipeline consists of viral inactivation, rendering samples safer to work with, followed by a sensitive 30-min isothermal detection reaction with a color-based red to yellow readout. Sensitivity can be further improved using a simple and inexpensive purification protocol. This pipeline can help address the shortage of testing capacity and can be run in a variety of settings.

The current severe acute respiratory syndrome coronavirus 2 (SARS-CoV-2) pandemic has had and will continue to have an enormous impact on society worldwide, threatening the lives and livelihoods of many. As the disease has spread, the need for rapid point-of-care diagnostic tools has become immense. Many efforts are currently underway to develop assays that can be used in a variety of settings (1). The ideal assay would require no specialized equipment and would have a rapid, easily read result. To that end, we have developed an assay based upon the reverse-transcription loop-mediated isothermal amplification (RT-LAMP) technique. To boost sensitivity, we also developed a sample preparation method that can be used as the first step for many different types of downstream assays. The protocol is simple, inexpensive, and rapid and utilizes reagents that are readily prepared in large quantities.

LAMP is a method of isothermal DNA replication that utilizes, in an accelerated format, six DNA oligos that hybridize with eight different regions of a target molecule (2, 3). Utilizing a strand-displacing polymerase and loops formed during this reaction, a fast amplification reaction occurs upon proper oligo binding to the desired target. Such reactions generate microgram quantities of DNA in a very short period of time at a single reaction temperature. Furthermore, although the strand-displacing polymerase has reverse transcriptase activity, a reverse transcriptase can be included to improve sensitivity within the reaction when detecting an RNA target (RT-LAMP), such as the SARS-CoV-2 RNA. LAMP assays have a variety of readouts due to the large amount of DNA generated, including fluorescence using an intercalating DNA dye, turbidity, or a drop in the pH if the reaction is minimally buffered (1, 4, 5). This change in pH, sufficient to cause a pH indicator dye to visibly change color, is the optimal method for a point-of-care LAMP-based diagnostic.

We designed and tested 11 sets of primers for the RT-LAMP assay and used the LAMP reaction reagents from New England Biolabs (NEB) for a colorimetric readout. These were tested relative to other primers recently published by NEB. An optimal set of primers directed toward a nonconserved region of the SARS-CoV-2 Orf1a gene was identified as being particularly sensitive without being prone to background signals. In addition to developing a robust RT-LAMP primer set, we also sought to optimize sample preparation in a way that would maximize sensitivity and render samples stable and safe for testing personnel. We explored the tolerance of the RT-LAMP reaction to detergents and chaotropic salts that might aid in the lysis of virions and purification of viral RNA genomes and messenger RNA. In addition, we optimized a very simple and rapid protocol based on the HUDSON method (6) for inactivating virions as well as endogenous RNases, adjusting the formulation to ensure its compatibility with a pH-based readout. This latter modification had the benefit of decreasing the temperature at which RNases are inactivated. These methods allow at least 5 µL of sample (nasopharyngeal [NP] swabs in saline/phosphate-buffered saline [PBS] or straight saliva) to be added to a reaction, to bring sensitivity to 10 to 50 RNA copies per microliter of sample. We also developed a simple and rapid process by which viral RNA can be purified and concentrated from 0.5 mL of collection media such that, when used with our Orf1a primer set, the limit of detection falls at least to 2 RNA copies per microliter. Unlike purification schemes used for the current Food and Drug Administration (FDA)-approved qRT-PCR−based test, this purification does not require a commercial kit. It binds nucleic acids to silica in the form of a suspension known as “glass milk” (7), which is readily available in industrial quantities at little expense. The overall cost of the inactivation and purification is approximately $0.07 per sample. Early clinical validation of these protocols with patient samples consisting of swabs in saline has shown that inactivation alone increases the sensitivity to >95% of samples with qRT-PCR Ct values of <30, and 85% sensitivity overall (8). Glass milk purification increases this sensitivity at least 10-fold. Furthermore, the specificity was found to be 100%, with no false positives reported (8).

## Results

### Sensitivity of HMS and NEB RT-LAMP Assays.

**Fig. 1.**
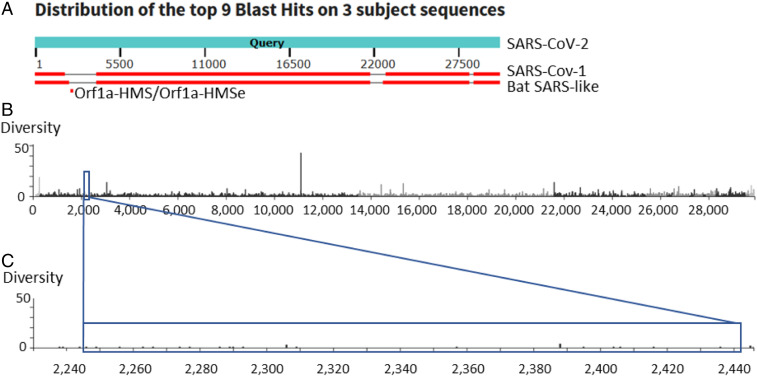
Sequence conservation of Orf1a-HMS target region between related coronaviruses and within SARS-CoV-2 isolates. (*A*) An alignment (blastn, megablast) of SARS, Bat SARS-like coronavirus isolate Rs4084, and the sequence detected by Orf1a-HMS/Orf1a-HMSe. (*B* and *C*) Measure of sequenced nucleotide mutations in a subset of global data provided by Nextstrain (nucleotide entropy), adapted from Nextstrain visual interface. Orf1a-HMS/Orf1a-HMSe target region (2245 to 2441) indicated. (*B*) Whole genome view. (*C*) Close-up of the target region.

**Fig. 2.**
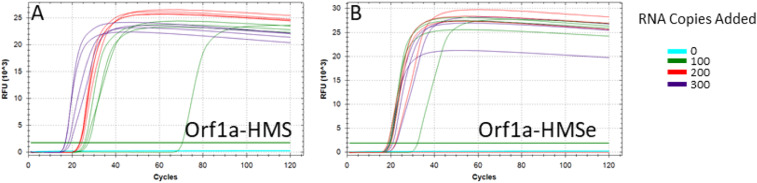
Initial sensitivity test of selected RT-LAMP primer sets. Fluorescent RT-LAMP reactions run for 120 30-s cycles at 65 °C: 0 (blue), 100 (green), 200 (red), or 300 (purple) control RNA copies included per reaction (*n* = 4). Assays performed were (*A*) Orf1a-HMS and (*B*) Orf1a-HMSe. RFU, relative fluorescence units.

We designed and tested 11 sets of LAMP primers for the SARS-CoV-2 genomes using V5 (https://primerexplorer.jp/e/), with the exception of the loop primers for Orf1a. PrimerExplorer could not find a set for this region of the genome, so loop primers for Orf1a-Harvard Medical School (Orf1a-HMS; *SI Appendix*, Table S1) were designed manually. Orf1a-HMS is in a region of SARS-CoV-2 that is not conserved with either SARS or Bat SARS-like coronavirus isolate Rs4084, two closely related coronaviruses (Fig. 1*A*). Despite this lack of conservation among coronaviruses, sequencing data from clinical SARS-CoV-2 isolates from around the world, accessed through Nextstrain (9), do not show that this target region is mutating more quickly than the rest of the genome (Fig. 1 *B* and *C*). Initial tests showed that Orf1a-HMS primers outperformed our other sets of primers. We then created Orf1a-Harvard Medical School Enhanced (Orf1a-HMSe, *SI Appendix*, Table S1) by modifying the forward inner primer (FIP) and backward inner primer (BIP) to include a “TTTT” linker between the F1c and F2 regions, as this has been reported to further improve the reaction (10). Orf1a-HMS and Orf1a-HMSe were tested using the NEB’s WarmStart LAMP Kit (NEB E1700) with a real-time fluorescence-based readout (Fig. 2). In this reaction scheme, each cycle represents 30 s at 65 °C. Ideally, a positive result will be read after 30 min, or the 60th cycle, a time point used by Zhang et al. (1). We used positive control RNAs from Twist Bioscience (Sku 102019), in 10-µL reactions in triplicate, including 0, 100, 200, or 300 viral RNA copies per reaction. As can be seen, both primer sets are capable of detecting viral RNA at low copy number. In order to further assess sensitivity, we ran repeated reactions using the same fluorescence-based readout with Orf1a-HMS, Orf1a-HMSe, and NEB Orf1a-C (*SI Appendix*, Fig. S1). For each, we ran 48 10-µL reactions with 200 viral RNA copies each and 48 10-µL reactions with no viral RNA added. As can be seen, both Orf1a-HMS and Orf1a-HMSe performed well, showing high amplification in 45 and 47 out of 48 reactions with 200 RNA copies, respectively. Furthermore, none of the reactions without viral RNA exhibited any amplification by 60 min. NEB Orf1a-C did not perform as well, as the time to amplification in the 200 RNA copy reactions was highly variable, with many not amplifying until just before or after the 30-min point. Furthermore, two reactions without viral RNA exhibited amplification, but we cannot rule out the possibility that these reactions, as sensitive as they are, were contaminated. These data suggest that Orf1a-HMS and Orf1a-HMSe are the more robust primer sets for this assay.

### Detergent Tolerance.

**Fig. 3.**
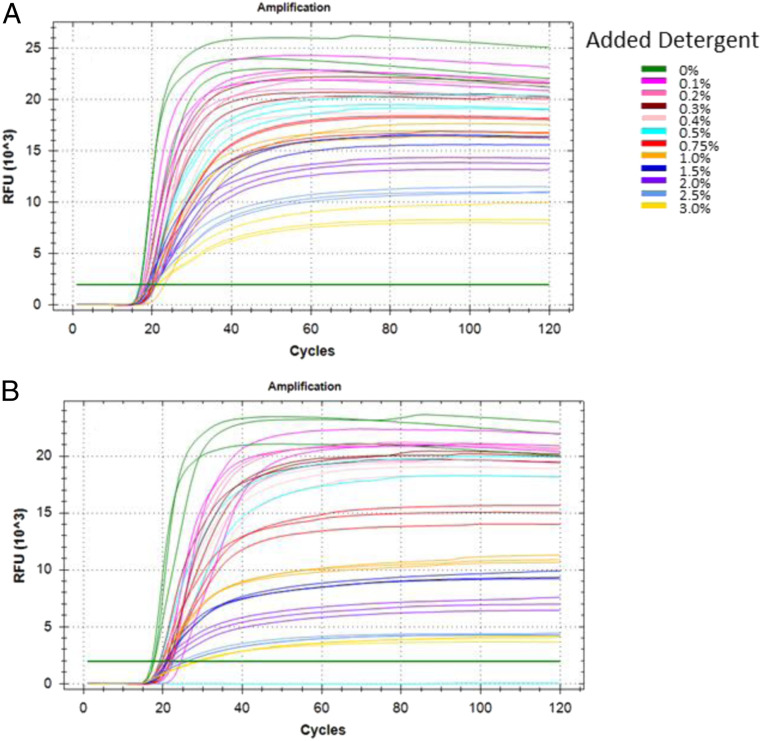
Assessment of RT-LAMP detergent tolerance. Fluorescent RT-LAMP reactions run for 120 30-s cycles at 65 °C. All reactions contain 500 control RNA copies, the Orf1a-HMS primer set, and 0 to 3% added (*A*) Tween20 or (*B*) TritonX100, as indicated.

In order to potentially improve the sensitivity of the RT-LAMP reaction when using patient samples, we hypothesized that an increase in detergent within the reaction might help to lyse virions, making their genomic RNA more accessible for reverse transcription and amplification. Using Orf1a-HMS and the same 10-µL fluorescent reactions as described above, we ran reactions with 500 viral RNA copies and differing amounts of added Tween20 or TritonX100. The reaction is quite tolerant of added detergents, and robust amplification could be seen up to at least 1.5% Tween20 and 1% TritonX100 (Fig. 3). Amplification could still be detected for both detergents up to 3%, but the reactions appeared to plateau at a lower level of fluorescence when detergent levels increased.

### Comparison of NEB and HMS Primer Sensitivity to Chaotropic Salts Using Colorimetric Readouts.

In order to optimize protocols that use chaotropic salts during purification, we tested the tolerance of the colorimetric RT-LAMP reactions to GuSCN and NaI, two such chaotropic salts, finding the reaction was tolerant of either up to 50 mM (*SI Appendix*, Fig. S2) (7, 11). While our final recommended protocol (below) does not utilize GuSCN (in part, due to production scale concerns as well as the danger of mixing GuSCN with bleach), we did develop a protocol that does use it, for situations where GuSCN is the chaotropic agent in use. We tested the effect of 50 mM GuSCN on the sensitivity of the RT-LAMP assay with various primer sets. This also served to directly compare the sensitivity of Orf1a-HMS, Orf1a-HMSe, NEB Gene N-A, and NEB Orf1a-C in the RT-LAMP reactions.

**Fig. 4.**
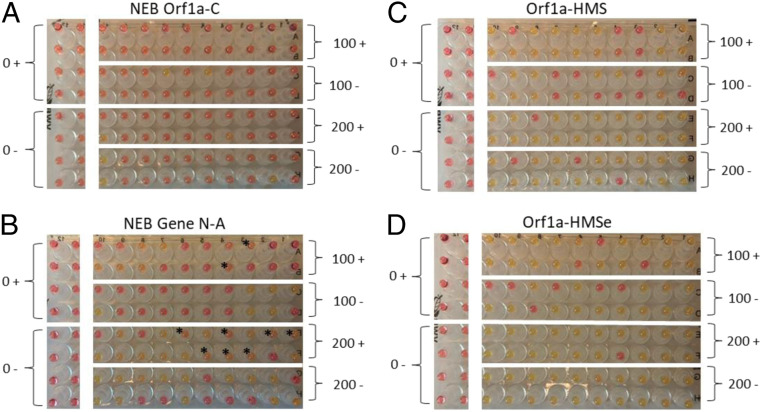
Assessment of GuSCN effects on sensitivity of colorimetric RT-LAMP assays. Colorimetric RT-LAMP reactions run with the number of control RNA copies (0, 100, or 200) noted. Reactions were run with 50 mM GuSCN (+) or without GuSCN (−) as noted. (*A*) NEB Orf1a-C primer set. (*B*) NEB Gene N-A primer set. (*C*) Orf1a-HMS primer set. (*D*) Orf1a-HMSe primer set. An * indicates reactions that were noticeably orange, but not completely yellow.

NEB Orf1a-C performed the worst with or without GuSCN (Fig. 4*A*), detecting 2/40 with 100 viral RNA copies and 5/40 with 200 viral RNA copies. This result was surprising, so we ran the experiment again, remaking all reagents including primer mixes and including four reactions of Orf1a-HMSe with 200 viral RNA copies as a plate control (*SI Appendix*, Fig. S3). The results were the same, with NEB Orf1a-C detecting 1/40 with 100 viral RNA copies, and 1/40 or 2/40 with 200 viral RNA copies (one of the 200 RNA copies reactions turned visibly orange, but not yellow). NEB Gene N-A performed better, detecting 11/40 to 13/40 with 100 viral RNA copies (depending on whether borderline orange reactions are considered to be positive) and 22/40 to 29/40 with 200 viral RNA copies (Fig. 4*B*). Orf1a-HMS and Orf1a-HMSe were much more sensitive. At 100 viral RNA copies, Orf1a-HMS and Orf1a-HMSe detected 26/40 and 31/40, respectively (Fig. 4 *C* and *D*). At 200 viral RNA copies, Orf1a-HMS and Orf1a-HMSe detected 36/40 and 39/40, respectively. Furthermore, all positive reactions were completely yellow, leaving no ambiguous orange reactions. None of the reactions without viral RNA resulted in a positive reaction for any of the assays tested (Fig. 4). There was also no difference in sensitivity between reactions that contained 50 mM GuSCN and those that did not when using the Orf1a-HMSe primers.

### Goals for Optimized Rapid Inactivation/Stabilization and Purification Protocol.

**Fig. 5.**
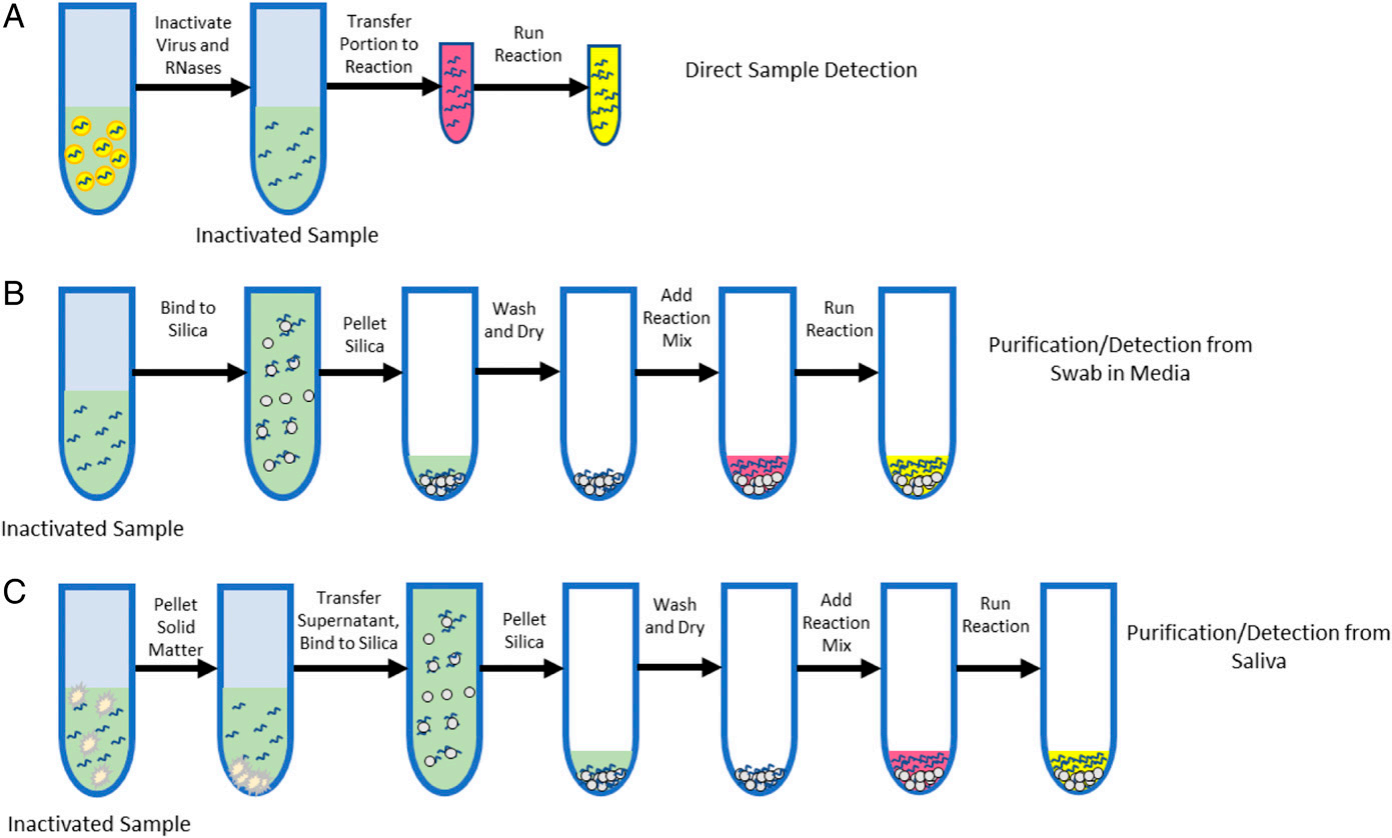
A simple and rapid sample inactivation and purification scheme. (*A*) A schematic depicting sample inactivation and direct RT-LAMP testing. (*B*) A schematic of the purification and testing procedure for swab samples postinactivation. (*C*) A schematic of the purification and testing procedure for saliva samples postinactivation.

The current sample collection methods used for SARS-CoV-2 testing require swabs to be placed in 2 mL to 3 mL of commercial collection media, such as Quest Diagnostics Viral Collection Media (VCM) (7). This method presents a serious challenge for RT-LAMP-based detection, as very little (no more than 1 µL) can be used in a 25-µL reaction, due to the presence of dyes and buffers in the VCM that would prevent visualization of a pH shift in a positive reaction (8). Other collection media, such as 0.9% saline, have less of an inhibitory effect. Finally, it is important to note that processing samples is not without risk, as such samples can contain infectious virus. Thus, we set out to design a protocol series that includes a rapid inactivation of virions in a variety of sample types while keeping the RNase inactivated. In addition, our goal was to have this protocol enhance sensitivity, while being compatible with both direct addition to RT-LAMP reactions and NaI-based purification (schematized in Fig. 5).

### Inactivation Protocol.

In order to quickly inactivate/lyse virions while also protecting their RNA from endogenous RNases, we employed a simple protocol, based on the HUDSON protocol (6), utilizing a shelf-stable reducing agent, Tris(2-carboxyethyl)phosphine (TCEP), the divalent cation chelator ethylenediaminetetraacetic acid (EDTA) and a brief period of heat at 95 °C. (However, lower temperatures may be sufficient; *SI Appendix*, Fig. S6.) With this protocol, 1/100th sample volume of a 100× TCEP/EDTA mixture (inactivation reagent) is added to the sample, which is then mixed and heated at 95 °C for 5 min. This protocol rapidly denatures proteins, utilizing the TCEP to reduce any disulfide bridges. Any divalent cations necessary for RNase activity are released from denatured proteins and sequestered by the EDTA, rendering any renatured RNases inert. We have found this sufficient to inactivate RNase activity; an added benefit is that reducing agents have been shown to reduce the viscosity of saliva and mucus (12). The resulting inactivated sample is fully compatible with direct addition to an RT-LAMP reaction, with at least 5 µL being tolerated. This protocol should be sufficient to inactivate any SARS-CoV-2 virions in the sample, rendering the sample much safer for downstream handling and transport (13, 14). Previously developed lysis methods for RNA viruses use a 95 °C lysis step, and Middle East respiratory syndrome-CoV is highly sensitive to even 1 min at 65 °C (6, 14).

**Fig. 6.**
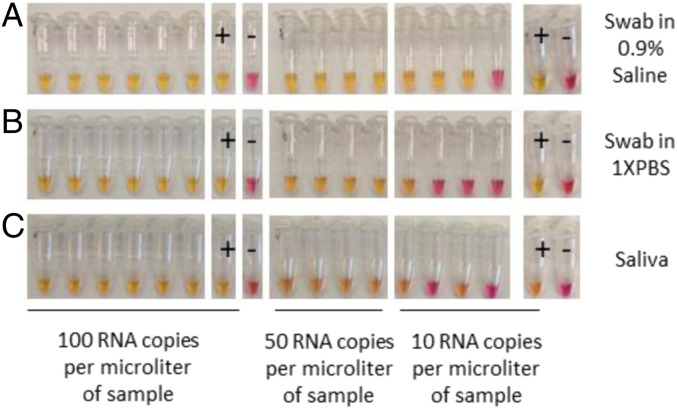
Sensitivity test with Orf1a-HMSe primer set following sample inactivation. Direct detection in reconstituted throat and nasal swabs in (*A*) saline, (*B*) 1× PBS, or (*C*) reconstituted saliva. Control RNA copies were spiked into samples during inactivation (concentration indicated). Negative control samples (−) had no RNA added. Following inactivation, 5 μL of sample was added to a colorimetric RT-LAMP reaction with Orf1a-HMSe primers. Positive control reactions (+) had an additional 1,000 control RNA copies added directly to the reaction.

Using this protocol, at least 5 µL of inactivated sample, including swabs in saline and 1× PBS, as well as straight saliva (without any other preparation), can be added to the reaction, allowing for robust detection at 50 RNA copies per microliter in the original sample (Fig. 6). This protocol can also be completed with nothing more than this inactivation reagent and a boiling water source, allowing for rapid sample inactivation and stabilization in a wide variety of settings. The inactivation leaves a sample at a slightly alkaline pH with 2.5 mM TCEP and 1 mM EDTA, so such inactivated samples will likely be compatible with a variety of nucleic acid detection tests, not just the RT-LAMP assay. We also have found dithiothreitol (DTT) can work in place of TCEP, with a minor change in the sodium hydroxide (NaOH) used, as DTT is not as acidic as TCEP. DTT is far less stable, however, so we chose TCEP as the optimal shelf-stable reagent.

### Purification Protocol.

In order to further increase the sensitivity of this test in a way that is inexpensive, accessible, and easily made to scale, we sought to optimize a purification protocol capable of concentrating viral RNA from a larger sample volume into a single reaction. We thus tested a very inexpensive and highly available silica particle suspension known as “glass milk” (7). This suspension is made by cleaning small silicon dioxide particles and suspending them in an equal volume of water. A single laboratory can prepare enough for tens of millions of purifications in a day at a cost of less than $45 per 1 million purifications. This suspension is the forerunner to the commonly used silica-based column purification kits used today. As with these columns, nucleic acids will bind to the silica in the presence of chaotropic salts, such as GuSCN or NaI (7, 11).

Our preferred protocol for purifying RNA relies on NaI, which was often used as the chaotropic salt of choice for purifying DNA from agarose gels using glass milk (7). In such protocols, three volumes of 6 M NaI was added to gel slices to melt the gel and bind the DNA to the glass milk. We found a final concentration of 2 M NaI was sufficient to concentrate control RNA genomes with glass milk. This is quite convenient, as using a smaller volume of a binding agent allows for a greater sample volume to be processed.

### Purification from Swabs in Saline/PBS.

**Fig. 7.**
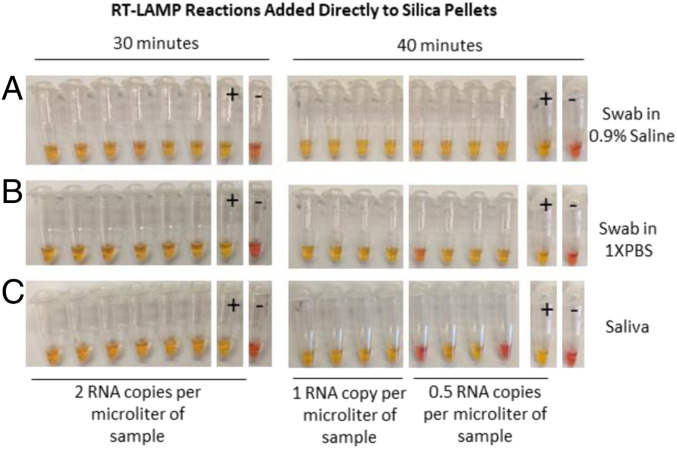
Sensitivity test with Orf1a-HMSe primer set following sample purification. Direct detection in reconstituted throat and nasal swabs in (*A*) saline, (*B*) 1× PBS, or (*C*) reconstituted saliva. Control RNA copies were spiked into samples during inactivation (concentration indicated). Negative control samples (−) had no RNA added. Following inactivation, samples were purified using glass milk with a centrifuge, and colorimetric RT-LAMP reaction with Orf1a-HMSe primers was added directly. Positive control reactions (+) had an additional 1,000 control RNA copies added directly to the reaction. Reactions were run for 30 min to 40 min at 65 °C, as indicated.

For samples that consist of swabs in saline or 1× PBS, one adds 1/2 volume of a NaI-based binding solution (6 M NaI, 2% TritonX100, 10 mM HCl) and 5 µL of glass milk to the sample following the previously described heat inactivation step (Fig. 5*B*). The sample is then mixed and incubated at room temperature for 10 min to allow the RNA to bind (the samples are inverted every 1 min to 2 min to keep the silica in suspension). The samples are then pulse spun in a tabletop microfuge, such as the VWR Galaxy Mini (or in a larger tabletop centrifuge at 2,000 × g), to pellet the silica, and the supernatant is poured off. A single wash with 80% ethanol is performed, with a final spin and use of a micropipette or fine-tip transfer pipette to remove the last traces of 80% ethanol. The pellet of silica particles with bound RNA is dried, either at room temperature or at 65 °C. Once dried, a 25-µL LAMP reaction can be added directly and the silica resuspended, at which point the RNA elutes into the reaction. The reaction can then be run directly, or the sample can be transferred to a different reaction vessel, with the silica particles included. As the reaction runs, the silica particles sink to the bottom of the tube and remain inert. When purifying from swabs in saline or 1× PBS, this allows for robust sensitivity down to at least one genome per microliter in the original sample (Fig. 7 *A* and *B*).

We have found that the protocol described above can be easily adapted to situations in which a centrifuge is not available. Following the binding of the RNA to the silica, samples can simply be allowed to sit undisturbed for 5 min to 10 min, allowing the majority of the silica to settle to the bottom of the tube; the supernatant can then be poured off. For this procedure, we included two washes with 80% ethanol to ensure removal of inhibitors, allowing 2 min for the silica to sink to the bottom of the tube between washes. We used a fine-tip transfer pipette to remove the final wash before drying the pellet and running the reaction as described above. While some silica particles are lost, particularly the smallest particles which do not settle easily out of solution, we were still able to achieve a sensitivity down to at least 1 RNA copy per microliter when purifying from 0.5 mL of swabs in saline of 1× PBS (*SI Appendix*, Fig. S4).

### Purification from Saliva.

We also sought to develop a protocol to purify viral RNA from saliva, as swab supplies could become limiting, and collection of saliva does not require the aid of a health care worker, as does the current NP swab protocol. We started with saliva inactivated by the inactivation protocol described above (Fig. 5*C*). Before adding NaI or glass milk, the samples are spun in a benchtop microfuge, such as the VWR Galaxy Mini, or another centrifuge, at 2,000 × g for 10 s to 15 s. This step causes the flocculant material in these samples to pellet, allowing the cleared sample to be poured into a fresh tube. Once this “preclearing” step is completed, the purification from the cleared supernatant can proceed exactly as described above for swab samples in media, allowing for a sensitivity down to at least 1 RNA copy per microliter when starting with 0.5 mL of saliva (Fig. 7*C*).

Unfortunately, we have been unable to perfect a protocol for purification from saliva that does not rely on a small centrifuge, although recent innovations, such as a handheld easily made “paperfuge,” may serve to overcome this problem (15). We tried allowing the flocculant material to settle by gravity for 30 min (during which much of it does settle to the bottom of the tube, but not all) and transferring the rest to a fresh tube, without success. We will continue to work on this, and invite others to do so as well, in order to make saliva-based purification protocols as accessible as possible.

### Purification from Commercial Collection Media.

We also optimized protocols for purifying from commonly used collection media, including Quest Diagnostics VCM and PrimeStore MTM. Viral RNA can be purified from VCM following the heat-based inactivation with TCEP and EDTA, after which the gelatin in the collection media crashes out of solution. The sample can be spun down in a centrifuge, and the cleared supernatant can be used for purification, with NaI binding solution supplemented with slightly more HCl. Samples in PrimeStore MTM can be treated in the same way as saline/PBS samples that have already been inactivated. Thus, 1/2 volume of the NaI binding reagent can be added directly, and purification can proceed. With both, comparable sensitivities were seen compared with the other sample types described above (*SI Appendix*, Fig. S5).

### RNA Stability Postinactivation and throughout Purification.

**Fig. 8.**
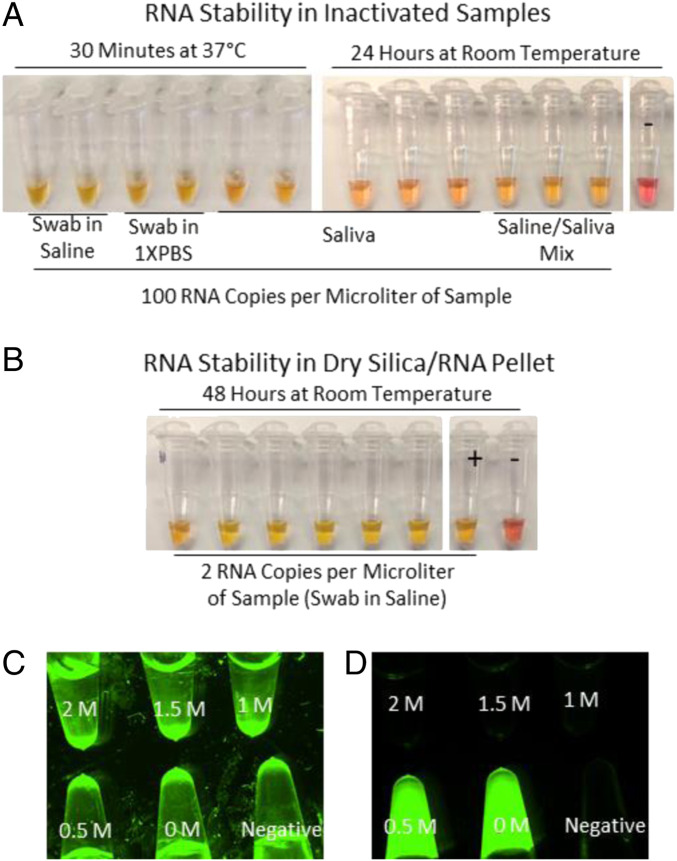
Evidence for RNA stability from inactivation through purification. (*A*) Swabs in saline, swabs in 1× PBS, saliva, or a 1:1 mixture of saliva and saline was inactivated and spiked with control RNA to 100 copies per microliter. After inactivation, samples were incubated for 30 min at 37 °C for 30 min or 24 h at room temperature, as indicated, before 5 μL was added to colorimetric RT-LAMP reaction mix with Orf1a-HMSe primers. (*B*) The 500-μL swabs in saline were purified using glass milk with a centrifuge. Dried RNA/silica pellets were left at room temperature for 48 h before adding colorimetric RT-LAMP reaction mix with Orf1a-HMSe primers. Negative control sample (−) had no RNA copies added to sample. Positive control reactions (+) had 1,000 RNA copies spiked directly into the reaction. (*C* and *D*) RNase activity from swabs placed directly into NaI solutions (concentration indicated). RNase Alert reactions incubated for 30 min at 37 °C; (*C*) bright field, (*D*) 488 nm fluorescence channel; fluorescence indicates RNase activity.

In order to verify that endogenous RNases are completely inactivated with these protocols, we inactivated samples of saliva, as well as swabs in saline or 1× PBS, and then added control viral RNA (100 copies per microliter) to probe for degradation. The inactivated samples with these control RNAs were incubated at 37 °C for 30 min, giving any residual RNase activity an opportunity to destroy control RNAs. When 5 µL of these samples were used in 25-µL RT-LAMP reactions with Orf1a-HMSe primers, all reactions returned positive (Fig. 8*A*). We also incubated similarly inactivated saliva and 1:1 saliva/saline mixtures for 24 h at room temperature before running the same reactions. Again, all reactions from samples with control RNA copies returned positive (Fig. 8*A*). We also found that RNA in the dried RNA/silica pellet is quite stable. Following purification, we incubated the dried RNA/silica pellet for 48 h at room temperature before running 25-µL RT-LAMP reactions with Orf1a-HMSe primers. All reactions from samples with control RNA copies added (to two RNA copies per microliter) returned positive (Fig. 8*B*). This indicates that RNases are completely inactivated with this protocol. We also tested different temperatures for inactivation. Incubation at 25 °C to 65 °C did not fully inactivate RNases (*SI Appendix*, Fig. S6 *A* and *B*). However, using our inactivation reagent, which is alkaline in order to be compatible with the pH-based colorimetric readout, RNase activity was abolished with inactivation at 75 °C. Samples inactivated with the alkaline inactivation reagent, at or above 75 °C (*SI Appendix*, Fig. S6 *C* and *E*). If control RNAs were added to such inactivated samples, and then incubated at 37 °C for 30 min, followed by incubation at room temperature for 24 h, they returned positive (*SI Appendix*, Fig. S6 *C* and *E*). However, when using an inactivation reagent at a neutral pH, inactivation at 95 °C was required. This suggests that RNases can be inactivated more easily in an alkaline solution, potentially reducing any window between viral lysis and RNase inactivation. RNase activity also was inhibited by the NaI concentration used during binding for purification (2 M) (Fig. 8 *C* and *D*).

## Discussion

In this report, we have presented evidence of a sensitive RT-LAMP assay for the SARS-CoV-2 virus made even more sensitive by a rapid and highly accessible inactivation and purification scheme. The scheme is compatible with current collection methods in which swabs are placed into a large volume of collection media, as well as samples of straight saliva. This inactivation and purification scheme and RT-LAMP assay are simple and fast, are inexpensive, and do not rely on specialized equipment. RT-LAMP reaction mixtures can be lyophilized, with the mix remaining stable for years at 4 °C or weeks at room temperature (16, 17). This will remove the cold-chain barrier for deployment to underresourced areas where cold shipping and frozen storage may not be feasible. Such lyophilized reaction formats would also allow for increased sample volumes to be added to each reaction.

The inactivation protocol rapidly inactivates both virions (13, 14) and endogenous RNases (6). This works both to stabilize viral RNA prior to detection and to render samples safer for downstream handling. Furthermore, this inactivation protocol should be compatible with most other nucleic acid detection assays, including the currently utilized FDA-approved qRT-PCR test. The alkaline inactivation solution used here also reduces the temperature required for inactivation, from 95 °C to 75 °C (*SI Appendix*, Fig. S6), which can increase the safety of handling the samples.

The silica particles used for purification are made from a crude silicon dioxide powder and can be prepared in enormous quantities very quickly. Furthermore, very little is needed for each purification (1 L is enough for 200,000 purifications). A single laboratory can easily make enough for millions of tests, allowing for institutions with basic equipment like centrifuges and autoclaves to generate enough supply to meet high demand. Each swab sample purification can be performed in minutes in a single tube without a centrifuge, using only two solutions. Multiple purifications can be processed in parallel, allowing for efficient purification by medical personnel in point-of-care institutions. Purified samples can be stored at room temperature following the glass milk purification procedure, allowing samples to be collected and stored prior to analysis in multiple types of assays.

These protocols can be used together in a single pipeline. Patient samples can first be inactivated and tested directly in an RT-LAMP assay with Orf1a-HMSe primers, with a sensitivity of at least 50 viral RNA copies per microliter, in 30 min. Samples showing negative results in this assay can then be directly used in the glass milk purification protocol and retested, increasing the sensitivity to 1 viral RNA copy per microliter. The flexibility afforded by the use of directly inactivated samples, or by the use of the purified samples, allows multiple schemes to be deployed. Furthermore, glass milk purification with gravity settling should be compatible with robotics pipetting platforms, with slow aspiration to leave behind the silica pellet during washes. Alternatively, larger particles of silica are available from the ceramics industry. If larger sizes are used, a larger volume of particles would need to be used to compensate for a reduction in the surface to volume ratio. However, the trade-off in terms of settling might be warranted for certain applications.

A sample can be processed initially using multiple sets of primers (either in the same reaction, in parallel reactions, or in sequential reactions) to ensure specificity and sensitivity. For example, if patient samples were tested separately with both Orf1a-HMSe and NEB N-A assays, a double positive result would be a more certain positive result. It is also possible that primer sets targeting genomic regions toward the 3′ end of the virus that are more abundant in subgenomic RNAs, such as the nucleocapsid gene, in infected cells would have increased sensitivity if those RNAs are found in patient samples (18). This would not be reflected in our RNA controls, which contained equal levels of all genomic regions. Furthermore, if a genomic mutation were to prevent one assay from returning a positive result, a second assay targeting a different region of the genome could still detect the viral RNA. It is also important that tests used for patient samples include an internal control, such as beta-actin used in the clinical validation of this assay (8), to ensure that RNA integrity in the sample is maintained and a negative result is not due to degradation.

When used with patient samples, Orf1a-HMSe performed very well, with inactivation greatly improving the limit of detection (8). Inactivation and use of the Orf1a-HMSe primers provided for a sensitivity of ∼85% of the currently used qRT-PCR assay, with robust sensitivity (>95%) for samples with Ct values under 30, corresponding to samples with ∼40 virions per microliter. Glass milk purification appeared to increase the sensitivity by at least 10-fold. Furthermore, the specificity was found to be 100% with no false positives. While not quite as sensitive as the currently used qRT-PCR method, the simplicity and speed in a wide variety of settings may still make this assay useful for infection control. Little is known about the range of viral concentrations that makes a person especially infectious to others. People with very low viral loads are most certainly less infectious, and thus their identification may not be critical to reducing viral spread. In addition, surveillance testing will likely be adopted to monitor populations fairly broadly and randomly (19, 20). Pooling without purification may suffice for detection of highly infectious individuals. However, since relatively large volumes can be concentrated by the glass milk procedure, samples can be pooled and purified by the glass milk protocol, likely without sacrificing sensitivity. This would enable surveillance testing without a great increase in cost (21).

We understand that many rapid tests are being developed daily and are reaching FDA approval. However, given the incredible demand, a variety of tests with different components from different industry sources are needed to address the immediate shortage of tests in the face of a sweeping pandemic. Each protocol serves an important function, with different tests having different requirements, different sensitivities, and varying expenses.

## Materials and Methods

### Orf1a-HMS/Orf1a-HMSe Primer Design.

The primary oligos for Orf1a-HMS/Orf1a-HMSe, F3, B3, FIP, and BIP primers, were designed by PrimerExplorer V5 (https://primerexplorer.jp/e/). The loop primers (LF and LB) were designed by hand, checking for appropriate melting temperatures using SnapGene software predictions.

### Oligos.

All oligos were ordered from IDT and resuspended in UltraPure water at a 100 µM concentration. Oligos were combined to make 100 µL of 10× primer mix as follows: 16 µL of FIP, 16 µL of BIP, 2 µL of F3, 2 µL of B3, 4 µL of LF, and 4 µL of LB, and brought to 100 µL with UltraPure water.

### RT-LAMP Reactions.

All RT-LAMP reactions were set up as described by NEB protocols (E1700 and M1800) and run at 65 °C. Fluorescence based reactions were run as 10-µL reactions in a Bio-Rad CFX96 thermocycler for 60 min monitored every 30 s for fluorescence in the SYBR channel. Colorimetric assays were run as 25-µL reactions for 30 min at 65 °C in an Eppendorf thermocycler, except for reactions following purification of samples with 0.5 to 1 viral RNA copies per microliter of sample, which were run for 40 min for improved color change. Colorimetric assays were imaged using a Pixel 2 smartphone with default settings.

### Control RNA.

All viral RNA sequences used in this study were purified RNA controls from Twist Bioscience (SKU 102019, 1 × 10^6^ RNA copies per microliter), diluted appropriately in nuclease free water. They were nonoverlapping RNAs representing fragments of the genome, as appropriate for each set of probes. To verify Twist RNA concentrations, the control RNAs were processed by RT and quantified using digital droplet PCR (ddPCR), using primers HMS Orf1e F3/B3 and NEB N-A F3/B3 for their genomic regions. Values obtained were 33% and 40%, respectively, of the values provided by Twist, assuming that the RT rate and ddPCR detection were 100%. As 100% is unlikely, these RT-ddPCR values were considered to be validations of the stated concentrations.

### Clean Reaction Setup.

All reactions were assembled and sealed prior to running in a dedicated clean room that was regularly decontaminated with bleach and had limited personnel access. Once reactions were run, the reaction tubes or plates were never opened again, to prevent postamplification contamination of future reactions.

### Solutions.

All solutions were created from molecular grade reagents. To make 5 mL of 100× inactivation reagent, first, 358 mg of TCEP-HCl (Millipore Sigma 580567) was dissolved in water to create 2.5 mL of a 0.5-M solution. Then 1 mL of 0.5 M EDTA, pH = 8 (ThermoFisher Scientific AM9260G) was added. Finally, 10 N NaOH and UltraPure water (ThermoFisher Scientific 10977015) was added to bring the final volume to 5 mL and the NaOH concentration to 1.1 to 1.15 N NaOH (1.1 N for use with swabs in saline of 1× PBS, 1.15 N for use with saliva with a pH of ∼6.5). For other collection media, the NaOH concentration will need to be optimized to ensure the pH of the final inactivated sample falls within an acceptable range such that the sample does not, upon addition, immediately cause the LAMP reaction to turn yellow or prevent the LAMP reaction from turning yellow upon successful amplification.

To make the NaI binding solution, 224.8 g of NaI (Millipore Sigma 793558) was dissolved in UltraPure water to a final volume of ∼230 mL. To this, 2.5 mL of 1 N HCl (made from 37% stock, Millipore Sigma 320331) and 5 mL of tritonX100 were added and mixed before bringing the volume to 250 mL with UltraPure water. Over time, this solution may turn somewhat yellow, presumably due to oxidation that results in the formation of molecular iodine. However, when added to an inactivated sample containing TCEP, this iodine is quickly reduced, rendering the solution colorless. This does not appear to affect the purification. Furthermore, this coloration can be inhibited by reducing the solution’s light exposure (e.g., wrapping the solution container in foil).

The 1× PBS was purchased as is (ThermoFisher 10010023), and 0.9% saline was created by bringing 1.54 mL of 5 M NaCl to 50 mL with UltraPure water.

### Glass Milk Preparation.

To prepare glass milk, 325 mesh silicon dioxide (Spectrum Chemicals, SI108) was combined with an excess volume of 10% HCl (∼3 N HCl) made from combining 37% HCl (Millipore Sigma 320331) and MilliQ water (Millipore) in a fume hood (dry silica powder should not be inhaled). After acid washing for 4 to 8 h at room temperature, silica was pelleted by spinning 2 min at 5,000 × *g*, and the supernatant was poured off. The pellet was resuspended in four pellet volumes of MilliQ water and then pelleted again. This wash step was repeated for a total of six washes. The pellet was then washed with four pellet volumes of 10 mM Tris HCl, pH = 8 (ThermoFisher Scientific AM9855G) and 1 mM EDTA (ThermoFisher Scientific 15575020), and pelleted. Finally, the pellet was resuspended in one pellet volume of 10 mM Tris HCl and 1 mM EDTA and autoclaved. This autoclave step is likely superfluous, however, as acid washes should render the beads free of contaminants. The resulting 50% glass milk slurry can be stored at room temperature. Before use, care must be taken to vigorously resuspend the particles as they begin to settle quickly.

### RNase Activity Determination.

RNase activity was tested using IDT’s RNaseAlert substrate (IDT 11-04-02-03). Briefly, the detection substrate (an RNA oligo with a fluor and quencher) was resuspended in UltraPure water at a 10 µM concentration. For each test, 5 µL of this substrate and 5 µL of 10× buffer were combined with 40 µL of a solution that was being tested for RNase activity. A test solution for testing RNase activity in NaI was created by submerging and vigorously agitating a cotton tip applicator (Puritan 806-WC) that had been swabbed thoroughly at the back of the throat, in 500 µL of the designated NaI solution. A positive control was created by submerging a swab in water, and a negative control had clean UltraPure water used without any additions. For testing the effects of inactivation temperature on RNase inactivation with a fluorescent readout, throat swabs were similarly placed in saline with 1× inactivation reagent, with a negative control consisting of this solution without the addition of a swab. These reactions were then incubated for 30 min or 60 min for NaI and inactivation tests, respectively, at 37 °C and imaged in bright field and 488 nm with a Leica stereoscope.

### Mock Samples.

Mock swab samples were created in both saline and 1× PBS. To simulate a typical swab collection, one NP and one oropharyngeal swab were submerged and agitated in 3 mL of either solution. For saliva collection, first the mouth was rinsed with water. After 30 min without eating or drinking, saliva was collected.

### Sample Inactivation and Direct RT-LAMP Testing.

All experiments with inactivated samples used either mock swab samples or saliva and RNA controls. To each sample, 1/100th volume of 100× inactivation reagent was added. The samples were then mixed and placed in a heat block set to 95 °C. After ∼2 min, by which point the samples reached 95 °C, positive control RNAs were added, and the remainder of the 5-min inactivation proceeded. Samples were then cooled on ice. For testing remaining RNase activity, samples, once cooled, were placed at 37 °C for 30 min before being placed back on ice. For RT-LAMP testing, 5 µL of sample was added to 20 µL of 1.25× colorimetric RT-LAMP mix containing the Orf1a-HMSe primer set. For testing the stability of samples inactivated at different temperatures, the inactivation was performed for 5 min at the indicated temperature. For inactivation with inactivation reagent at a neutral pH, 100× inactivation reagent was prepared with 0.75 M NaOH. Following inactivation, just before adding sample to the reaction, 1/100th volume of 0.35 M NaOH was added to bring the pH of the sample to the proper alkaline range needed for the colorimetric readout.

### Sample Purification—with Centrifuge.

All purification experiments used 500 µL of sample (either swabs or saliva) and were performed in 1.5-mL tubes (Fisher Scientific 14-222-155) whose conical shape made retention of the silica pellets very effective. Samples were inactivated as described above and cooled. For saliva purifications, these samples were spun at 2,000 × *g* in a VWR Galaxy Mini centrifuge for 10 s to 15 s, and the cleared supernatant was transferred to a fresh tube for purification. This step was omitted for swab samples. Then 250 µL of NaI binding reagent was added along with 5 µL of glass milk (these could be combined beforehand into a master mix format, 255 µL of which was added to each). The tubes were then mixed by inversion and incubated at room temperature for 10 min; they were inverted every 2 min to resuspend the silica. Samples were then spun for 2 s to 3 s at 2,000 × *g* to pellet the silica, and the supernatant was poured off. Then 700 µL of 80% ethanol was added, and the tubes were inverted two or three times to wash (the pellet need not be resuspended). Samples were spun again for 2 s to 3 s, and the supernatant was poured off. Samples were spun for a final time to bring all residual 80% ethanol to the bottom. A micropipette or a fine-tipped transfer pipette (such as Thomas Scientific 232-11) was used to remove the residual solution from the pellet. The pellet was then completely air dried (until it resembled dry parchment), leaving the tubes open at room temperature or in a 65 °C heat block for faster drying. Then 25 µL of 1× colorimetric RT-LAMP mix with Orf1a-HMSe primers was added, and the pellet was resuspended by pipetting or flicking. The reaction was transferred to a 0.2-mL PCR tube before running at 65 °C for 30 min (this tube format makes for easier imaging).

### Sample Purification—without Centrifuge.

Purification using only gravity to pellet the silica particles was only successful using swabs, not saliva. Five hundred microliters of sample was inactivated, and RNA was bound to the silica with the NaI binding reagent as described above for use with a centrifuge. Samples were then allowed to sit undisturbed for 5 min to 10 min to allow the silica to settle out, and the supernatant was poured off (some small particles remain in the supernatant which will be cloudy, but a significant amount of silica settled to the bottom of the tube). Then 700 µL of 80% ethanol was added, and the tubes were inverted two or three times to wash (the pellet need not be resuspended). Samples were allowed to sit for 2 min to 3 min, and the supernatant was poured off. An additional 700 µL of 80% ethanol was added, and samples were allowed to sit for 2 min to 3 min. The supernatant was then removed with a fine-tipped transfer pipette moderately slowly (over 2 s to 3 s) to leave as little 80% ethanol as possible. The pellet was then completely air dried (until it resembled dry parchment) leaving the tubes open at room temperature or in a 65 °C heat block for faster drying. Reactions were then run as described above for purifications with a centrifuge.

### Sample Purification with Commercial Collection Media.

Viral RNA was spiked directly into clean samples of Quest Diagnostics VCM or PrimeStore MTM. For VCM samples (750 µL), 1/100th volume of 100× inactivation reagent, described above, was added, and samples were heated at 95 °C for 5 min and cooled. VCM samples were then spun at 10,000 × *g* in a centrifuge for 15 s to pellet the gelatin at the bottom of the tube, and the cleared supernatant was transferred to a fresh tube (2/3 the original sample volume, 500 µL). One-half of this cleared supernatant volume of the NaI binding solution, with an additional 12.5 mM HCl, was then added, along with 5 µL of glass milk. Purification was then performed with a centrifuge as described above. For samples in PrimeStore MTM, no inactivation was performed. One-half of the sample volume of the NaI binding solution and 5 µL of glass milk were added, and purification was then performed with a centrifuge as described above.

## Supplementary Material

Supplementary File

## Data Availability

All study data are included in the article and *SI Appendix*.
