# Supplementary material for: SARS-CoV-2 detection using isothermal amplification and a rapid, inexpensive protocol for sample inactivation and purification
Source: Proc Natl Acad Sci U S A. 2020 Sep 8;117(39):24450–8. doi: 10.1073/pnas.2011221117 (PMC7533677; doi:10.1073/pnas.2011221117)
Supplement: Supplementary File [file pnas.2011221117.sapp.pdf]

Orf1a-HMS_F3	CGGTGGACAAATTGTCAC
Orf1a-HMS_B3	CTTCTCTGGATTTAACACACTT
Orf1a-HMS_LF	TTACAAGCTTAAAGAATGTCTGAACACT
Orf1a-HMS_LB	TTGAATTTAGGTGAAACATTTGTCACG
Orf1a-HMS_FIP	TCAGCACACAAAGCCAAAAATTTATCTGTGCAAAGGAAATTAAGGAG
Orf1a-HMS_BIP	TATTGGTGGAGCTAAACTTAAAGCCCTGTACAATCCCTTTGAGTG
Orf1a-HMSe_FIP	TCAGCACACAAAGCCAAAAATTTAT**<u>TTTT</u>**CTGTGCAAAGGAAATTAAGGAG
Orf1a-HMSe_BIP	TATTGGTGGAGCTAAACTTAAAGCC**<u>TTTT</u>**CTGTACAATCCCTTTGAGTG

Supplemental Table 1. Oligo Sequences used for Orf1a-HMS and Orf1a-HMSe Primer Sets

Both Orf1a-HMS and Orf1a-HMSe primer sets use the same F3, B3, LF, and LB oligos as shown. Orf1a-HMSe uses its own FIP and BIP oligos which are identical to those used by Orf1a-HMS with the exception of 4 thymidine residues inserted in the middle (underlined and bold).

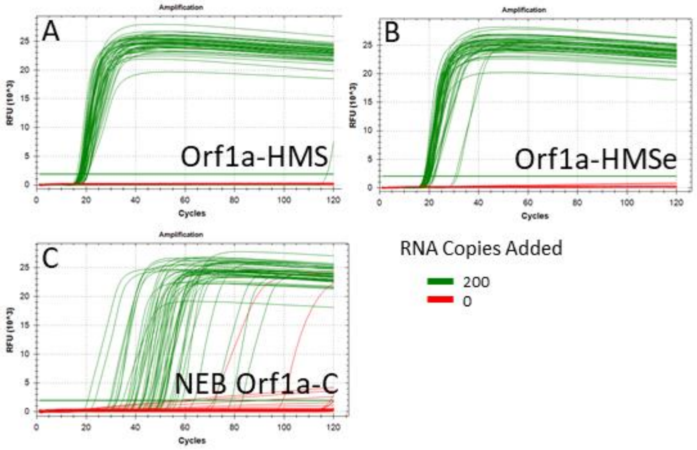

Supplemental Figure 1. Repetitions of Low RNA Copy Number Reactions

Fluorescent RT-LAMP reactions run for 120, 30 sec cycles at 65°C. 200 (green) or zero (red) control RNAs included per reaction (n = 48). Primer Sets used were A – Orf1a-HMS; B - Orf1a-HMSe; C - NEB Orf1a-C. RFU – Relative Fluorescence Units.

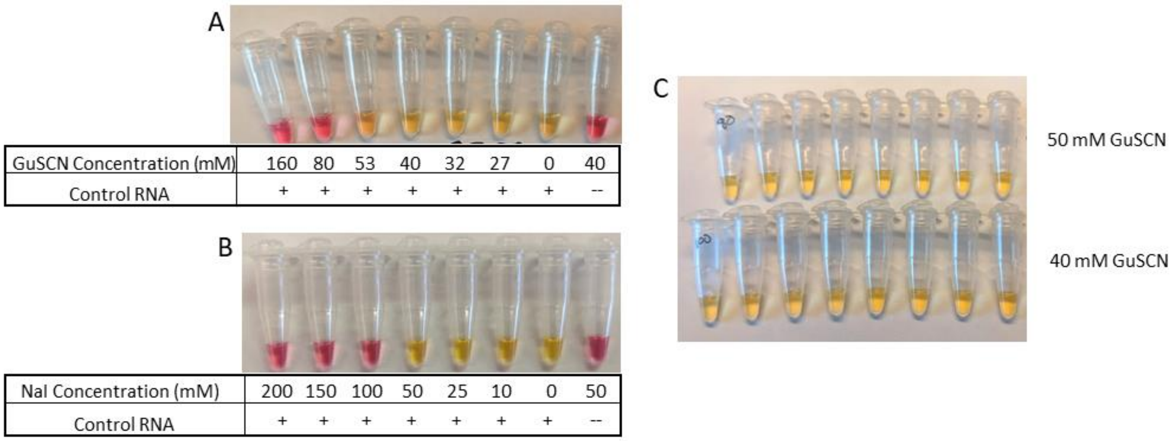

Supplemental Figure 2. Assessment of RT-LAMP GuSCN Tolerance

Colorimetric RT-LAMP reactions used the Orf1a-HMSe primer set. A - B – Reactions contained indicated GuSCN (A) or NaI(B) concentration and 500 control RNA copies (+) or 0 control RNA copies. C - Repeat reactions containing the indicated GuSCN concentration (50 mM or 40 mM) and 500 control RNA copies.

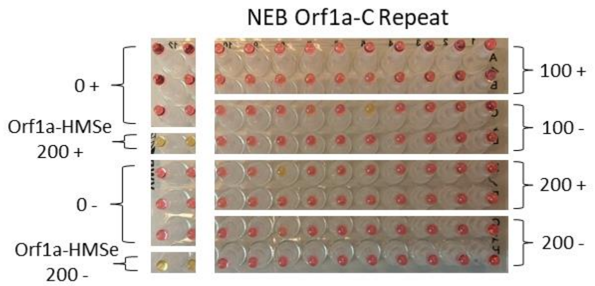

Supplemental Figure 3. Repeat Run of NEB Orf1a-C Primer Set

Colorimetric RT-LAMP reactions run with the number of control RNA copies (0, 100, or 200) noted. Reactions were run with 50 mM GuSCN (+) or without GuSCN (-) as noted. All reactions except the four noted used NEB Orf1a-C primer set. Four indicated reaction used Orf1a-HMSe primer set as a plate control.

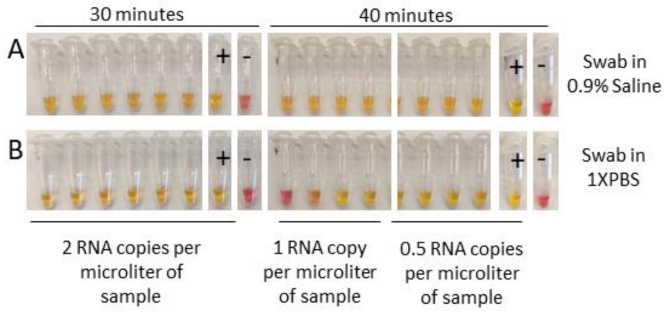

Supplemental Figure 4. Sensitivity Test with Orf1a-HMSe Primer Set Following Sample Purification without a Centrifuge

Direct detection in reconstituted throat and nasal swabs in saline (A) or 1XPBS (B). Control RNA copies were spiked into samples during inactivation (concentration indicated). Negative control samples (-) had no RNA added. Following inactivation, samples were purified using glass milk without a centrifuge, and colorimetric RT-LAMP reaction with Orf1a-HMSe primers was added directly. Positive control reactions (+) had an additional 1000 control RNA copies added directly to the reaction. Reactions were run for 30-40 minutes at 65C, as indicated.

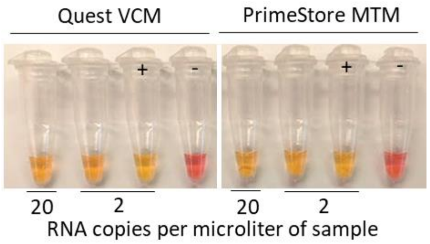

Supplemental Figure 5. Purification from Commercial Collection Media

750 µl of Quest VCM or 500 µl of PrimeStore MTM was spiked with control RNA copies to the indicated concentration and glass milk purified with a centrifuge, and colorimetric RT-LAMP reaction with Orf1a-HMSe primers was added directly. Negative control sample (-) had no RNA copies added to sample. Positive control reactions (+) had 1000 control RNA copies spiked directly into the reaction.

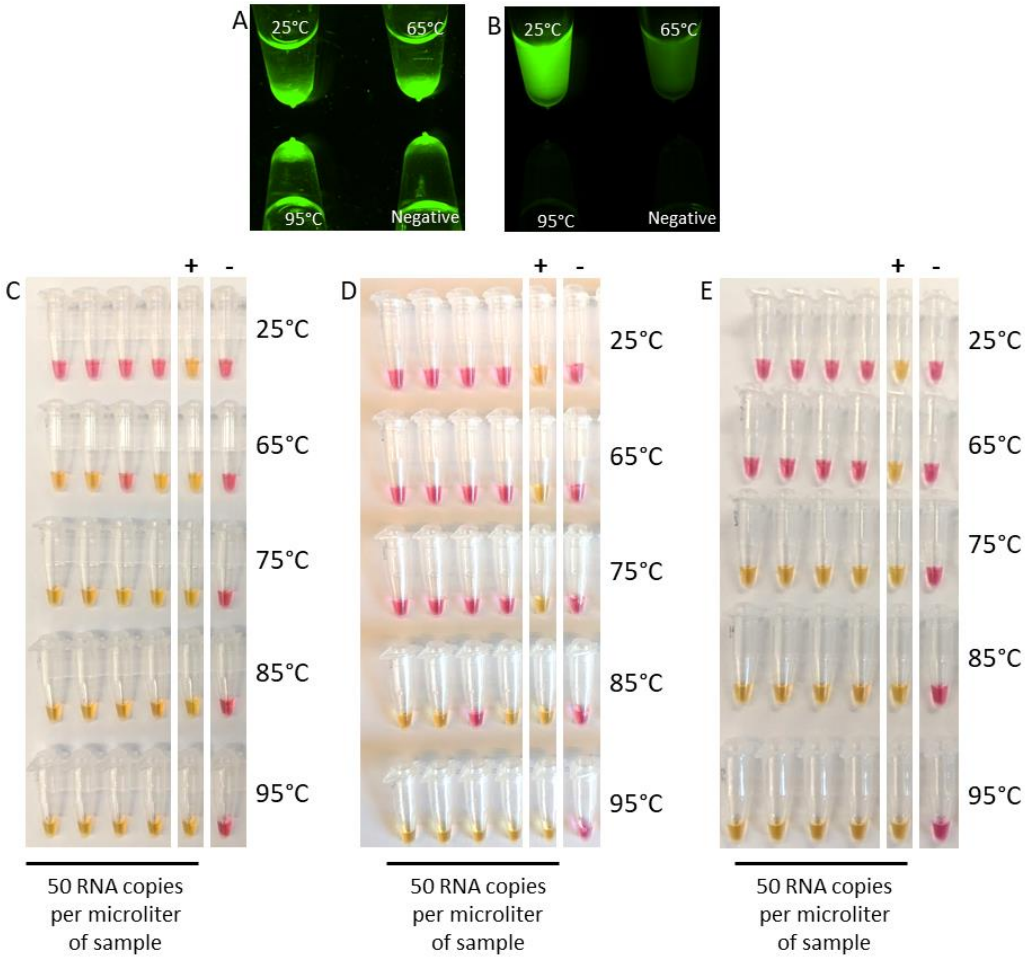

Supplemental Figure 6. Temperature Dependence of Sample Rnase inactivation

RNase activity from swabs in saline following a five-minute inactivation at the indicated temperature. RNase Alert reactions incubated for 60 minutes at 37°C. A– Brightfield. B– 488 nm fluorescence channel, fluorescence indicates RNase activity. C-D 25 µl colorimetric RT-LAMP reactions with HMS Orf1e primers with 5 µl of a swab in saline inactivated at the indicated temperature (25°C - 95°C) with alkaline (C and E) or neutral (D) inactivation reagent and spiked with 50 RNA copies per microliter or none (-) after inactivation. C-D - Before being added to RT-LAMP reaction, samples were incubated for 30 minutes at 37°C (C and D). E – Same samples used in C, incubated an additional 24 hours at room temperature before being added to RT-LAMP reaction.
